# Fistula Between the First Obtuse Marginal Branch of the Left Circumflex and the Left Ventricular Cavity: A Rare Anomaly

**DOI:** 10.7759/cureus.13316

**Published:** 2021-02-12

**Authors:** Arish Maknojia, Yuri Pride, Abhijit Ghatak, Jin Lee

**Affiliations:** 1 Internal Medicine Department, Northside Hospital Gwinnett, Lawrenceville, USA; 2 Cardiology Department, Cardiovascular Group, Lawrenceville, USA; 3 Cardiology Department, Cardiovascular Clinic of North Georgia, Lawrenceville, USA

**Keywords:** coronary cameral fistula, first obtuse marginal branch of left circumflex artery, left ventricle, incidental radiological finding, brief review

## Abstract

Coronary-cameral fistulae (CCF) are rare, frequently incidental findings uncommonly noted during routine coronary angiography. They are nearly always congenital and are sometimes associated with other cardiac malformations. They can also be acquired due to trauma or chronic inflammation. These fistulae most commonly originate from the right coronary artery. The site of termination is usually the right ventricle (RV) and rarely the left ventricle (LV). Though nearly always asymptomatic and clinically insignificant, depending on their size and pressure gradient between communicating sites and terminating area, CCF can lead to pulmonary hypertension, LV dysfunction, and myocardial infarction. We describe the case of a 55-year-old woman who presented with worsening dyspnea and lower extremity edema. Transthoracic echocardiography demonstrated an ejection fraction of 55% with an RV systolic pressure of 67 mmHg. Right heart catheterization was performed to formally diagnose pulmonary hypertension and left heart catheterization was performed concurrently. This demonstrated a fistula between the first obtuse marginal branch of the left circumflex artery to the LV cavity. In this report, the authors provide a brief review of the presentation, diagnosis, complications, and management of CCF.

## Introduction

Coronary artery anomalies occur in less than 1% of the general population. Coronary-cameral fistulae (CCF) are the abnormal vascular connections between coronary arteries and cardiac chambers. Abbott described coronary artery fistulae emptying into a cardiac chamber during autopsy studies in 1908 [1]. CCF can be congenital or acquired. They were previously thought to occur secondary to severe coronary atherosclerosis due to aberrant neovascularization in which collateral vessels inadvertently terminate into a cardiac chamber [2].

The clinical significance of these fistulae depend on the location and size. Small fistulae are usually silent and are typically identified as incidental findings on angiography. Large fistulae are diagnosed due to symptoms and its complications. The most frequent site for the fistula is between the right coronary artery and the right ventricle (RV) and rarely left ventricle (LV). We are presenting a case of a woman, who was diagnosed with rare communicating fistula between the first obtuse marginal branch of the left circumflex artery and the LV.

## Case presentation

The patient was a 55-year-old African American female with systemic lupus erythematous, interstitial lung disease on chronic oxygen, history of pulmonary embolism, heart failure with preserved ejection fraction, and pulmonary arterial hypertension who presented with dyspnea and orthopnea. Her home medications included hydroxychloroquine, mycophenolate mofetil, prednisone, furosemide, and spironolactone. Family history included congestive heart failure due to hypertension suffered by her sister, stroke by her father, and ovarian cancer by her mother. There was no history of congenital heart disease in the family. Social history included no tobacco abuse and occasional alcohol use.

On physical examination, her temperature was 97°F, heart rate was 79 beats per minute, blood pressure was 130/93 mmHg, respiratory rate was 20 breaths per minute, and her oxygen saturation was 95% on 4 L oxygen. Heart sounds were audible with regular rhythm and a 2/6 systolic murmur heard best at the left lower sternal border. Lung sounds demonstrated inspiratory crackles from mid-to-lower bases bilaterally.

Pertinent lab values revealed NT-proBNP of 2,356 and negative troponins. Electrocardiogram (ECG) demonstrated normal sinus rhythm with RV hypertrophy and non-specific ST-T wave abnormalities. Computed tomography (CT) angiogram of the chest demonstrated peripheral cystic lung changes bilaterally, cardiomegaly with enlargement of RV and right atrium along with straightening of interventricular septum with no evidence of pulmonary embolism, and enlargement of the pulmonary artery. The patient was started on furosemide, spironolactone, budesonide/formoterol, and methylprednisolone. Pulmonology and cardiology were consulted. ECG demonstrated normal LV systolic function with an ejection fraction of 50-55% with grade I diastolic dysfunction, severely dilated RV with decreased function, and moderate-to-severe tricuspid regurgitation. Estimated peak RV systolic pressure was 67 mmHg, reflective of severe pulmonary hypertension. Three months prior, her estimated RV systolic pressure was 39 mmHg.

The patient’s worsening dyspnea was attributed to progression of her interstitial lung disease, and the lung transplant service was consulted for further workup. The patient gradually improved during the hospital course, and she was discharged home with outpatient cardiology follow-up to perform a right and left heart catheterization to confirm the diagnosis of pulmonary hypertension and evaluation for lung transplantation.

**Figure 1:**
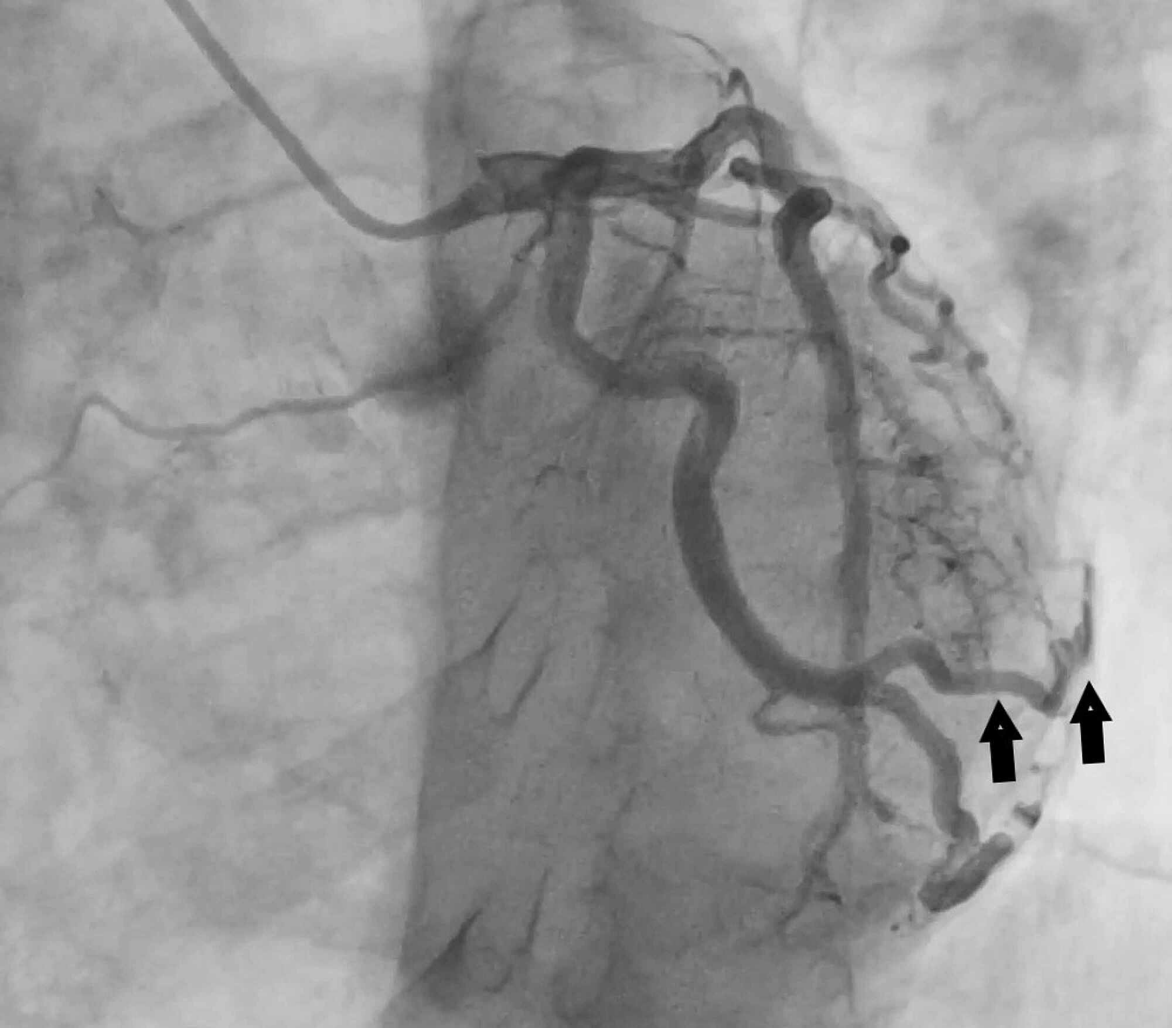
Left coronary angiogram demonstrating fistula between the first obtuse marginal branch of the left circumflex artery and LV cavity (arrow head). LV, left ventricle

**Figure 2:**
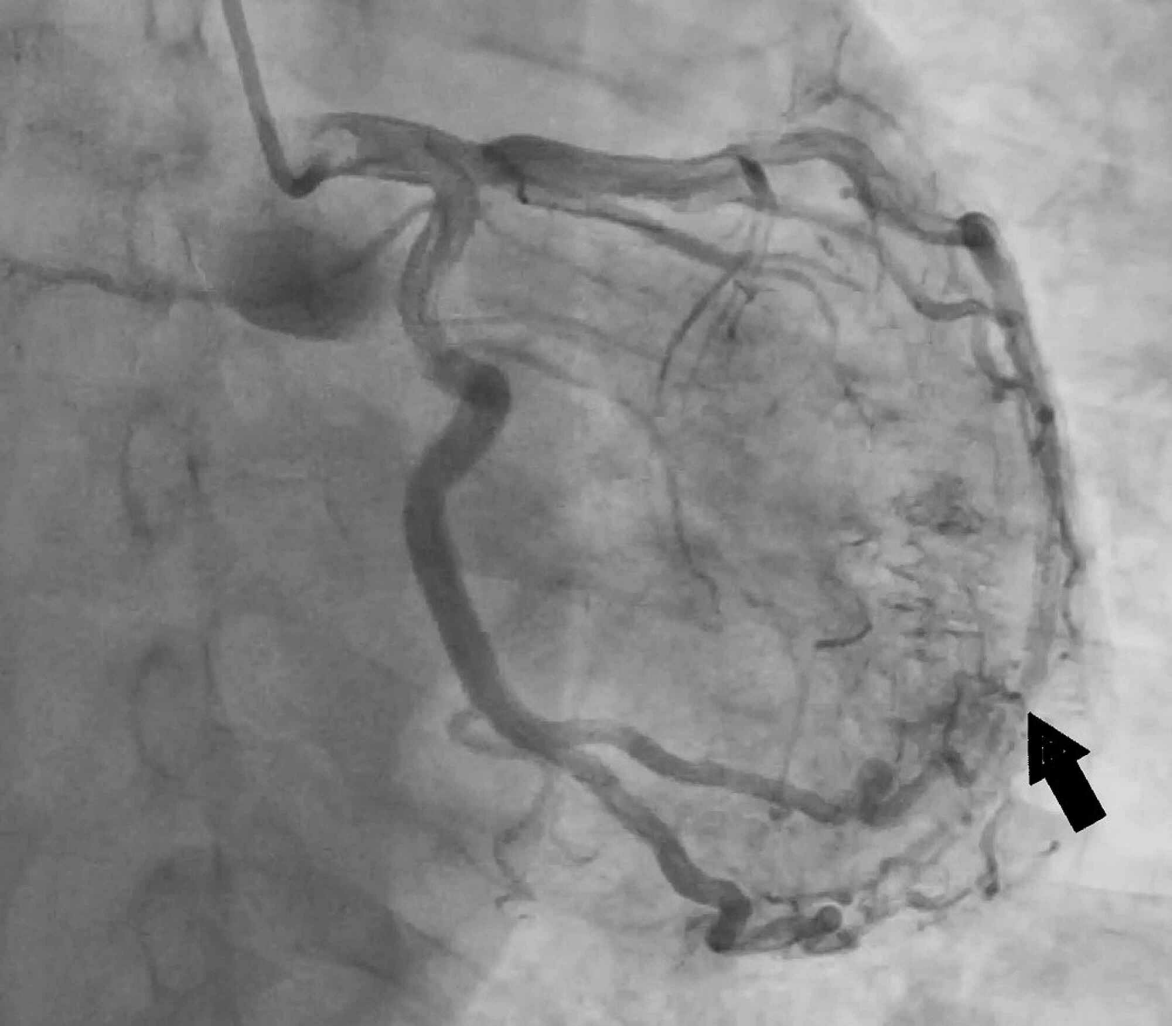
Another view of the left coronary angiogram re-demonstrating the fistula between the first obtuse marginal branch of the left circumflex artery and LV. LV, left ventricle

Outpatient right heart catheterization confirmed pulmonary artery pressures of 71 mmHg. Left heart catheterization demonstrated mid single-vessel atherosclerotic disease. However, there was a coronary fistula from the first obtuse marginal branch of the left circumflex artery to the LV (Figures 1, 2). The LV end diastolic pressure was 12 mmHg with an ejection fraction of 55% with no wall motion abnormalities. Because the patient was asymptomatic, no further intervention was performed.

Patient presented after one week with acute hypoxic respiratory failure due to multi-focal pneumonia requiring intubation and subsequently developed pulseless electrical activity cardiac arrest with unsuccessful cardiopulmonary resuscitation.

## Discussion

CCF is a rare anomalous connection between a coronary artery and a cardiac chamber. Apart from a cardiac chamber, coronary artery fistulae can also communicate with a major vessel (venae cava, pulmonary artery, veins, or coronary sinus) [3]. The incidence of coronary artery fistula is 0.1% to 0.2% in all patients undergoing coronary angiography [4]. CCF may be single or multiple and occur between one or more coronary arteries, bypassing the myocardial capillary bed [5]. During early fetal development, sinusoids nourish the primitive myocardium, which is connected to the primitive tubular heart. Persistent sinusoids that fail to regress later during adulthood may contribute to a congenital fistulous communication between the coronary arteries and the cardiac chambers [6].

Congenital coronary fistula can occur as an isolated incidental finding or can be associated with other congenital heart abnormalities such as pulmonary stenosis or atresia with an intact interventricular septum, pulmonary artery branch stenosis, tetralogy of Fallot, coarctation of the aorta, hypoplastic left heart syndrome, and aortic atresia [3,7]. Rarely, coronary artery fistulae can be acquired secondary to traumatic injury, cardiac surgery, percutaneous coronary intervention, endomyocardial biopsy, pacemaker implantation, or chest irradiation [6-8]. Several diseases, such as coronary vasculitis and myocardial infarction, can also lead to coronary arterial fistulae during the chronic phases [6]. CCF commonly originate from the right coronary artery (55%) but can also originate from the left coronary artery (35%) or bilaterally (5%) [5]. CCF most commonly terminate in the RV (41%), followed by the right atrium (26%) and the left atrium and LV (3%-5%). Clinical implication of CCF depends on the patient’s age, presence of symptoms and hemodynamic significance of the anomaly which relies on fistula size, pressure gradient, and resistance between the coronary artery and terminating chamber. Therefore, it is crucial to know about the origin and terminating site of these fistulas. Spontaneous closure of the fistula is rare, and has been is reported in 1%-2% of the cases; thus, regular follow-up is suggested [6].

Chest X-ray and ECG are not helpful in the diagnosis of CCF. Two-dimensional and color Doppler echocardiography may reveal the dilated coronary artery and color mapping may reveal the site of drainage. However, it is difficult to delineate the detailed anatomy of the fistula. Thus, cardiac catheterization remains the modality of choice for defining the size, anatomy, number, origin and termination site, and flow [8]. CT angiography and magnetic resonanca angiography can also help delineate the course and size of CCF [6].

Most fistulae are small and do not cause any signs or symptoms but can present with a continuous murmur. If the fistula connects to the LV only, an early diastolic murmur may be heard [7]. These asymptomatic fistulae are sometimes treated with anti-platelet therapy and/or antibiotics, though there are no guidelines to help define therapy [6]. Patient with such fistulae should get regular follow-up as smaller fistula can grow with age. Untreated hemodynamically significant fistulae may result in symptoms in 19% of the patients under the age of 20 and 63% of the patients over the age of 20. Larger fistulae can cause coronary artery steal, resulting in myocardial ischemia distal to the fistula causing angina and/or dyspnea [8]. Myocardial ischemia has been demonstrated on treadmill test in patients with coronary artery fistulae [9]. Acute myocardial infarction has also been reported due to coronary steal phenomenon by CCF [10]. Moreover, a CCF into a right heart chamber, thus causing a left-to-right shunt, has been to shown to lead to increased right heart volume and pulmonary hypertension [6]. A CCF into the left heart chamber (i.e., left-to-left shunt) leads to large left heart volume and increased LV pressure, causing LV hypertrophy and dysfunction. Patients can present with syncopal episode [11]. Other complications associated with CCF include aneurysmal formation, pericardial effusion, cardiac failure, atrial fibrillation, supraventricular and ventricular arrhythmias, bacterial endocarditis, thrombosis and/or embolism, rupture causing hemopericardium, sudden cardiac death, valvular regurgitation due to papillary muscle abnormality, and premature atherosclerosis [5-6,8].

Hemodynamic significant fistulae may be treated with either catheter closure or surgical repair. The indications for percutaneous transcatheter closure include proximal fistula origin, single drain site, no tortuous vessel with distal portion of fistula accessible with the closure device, extra-anatomic termination of fistula away from normal coronary arteries, older patients with high risk of perioperative complications, and absence of concomitant cardiac disorders [6]. Various percutaneous catheter techniques have been employed, including Gianturco coils, interlocking detachable coils, detachable balloons, polyvinyl alcohol foam, double umbrellas, the Amplatzer duct occluder, and the Amplatzer vascular plug. Risks of fistula closure with these devices include myocardial ischemia, migration of coils or discs to extra-coronary vascular structures or within the coronary artery branches, incomplete occlusion of the shunt if the coil is placed distal to the branch point, presence of foreign body increasing the risk of infective endocarditis, and catheter-related complications such as coronary artery vasospasm, dissection, and perforation [8,12,13].

The indications for surgical ligation of CCF include large symptomatic fistula with high fistula blood flow, multiple communications and draining sites, tortuous and aneurysmal fistulous artery, and need for simultaneous distal bypass and large vascular branches that can be accidentally embolized [6]. According to the American College of Cardiology and American Heart Association guidelines, there has always been a controversy regarding the management options to choose [4]. Surgical methods of closure are associated with low mortality and morbidity. However, there is a risk of myocardial infarction postoperatively because of low flow in the dilated coronary artery proximal to fistula closure [14]. Moreover, there is also a risk of recurrence of the fistula. Despite good results with surgery, percutaneous closure has become the method of choice [8].

Our patient had a CCF originating from first obtuse marginal branch of the left circumflex artery terminating into the LV with no hemodynamic significance with normal LV function, sustained ejection fraction, and normal LV end diastolic pressure. Moreover, the patient had other significant co-morbidities that required immediate attention without any further delay.

## Conclusions

CCF is a rare congenital anomaly usually found incidentally. Its clinical course ranges from no symptoms to serious complication including myocardial infarction and heart failure. Definitive diagnosis is with left heart catheterization, revealing originating and terminating site of the fistula. As no proper guidelines are available regarding the choice of appropriate treatment due to rare presentation, the treatment course is usually dependent on patient factors. Multi-disciplinary team involvement is necessary to guide further treatment in hemodynamic significant fistulas.
